# Perceived Clinical Barriers to Employment for Males with Spinal Cord Injury in Saudi Arabia

**DOI:** 10.3390/ijerph19084747

**Published:** 2022-04-14

**Authors:** Ahmad H. AlWashmi, Ahmad Zaheer Qureshi, Sami Ullah, Saeed Bin Ayaz, Nourah Hamad AlKeaid, Hind Miqad Alotaibi

**Affiliations:** 1 Department of Orthopedic Surgery, College of Medicine, Qassim University, Buraydah 52571, Saudi Arabia; 2 Department of Physical Medicine and Rehabilitation, King Fahad Medical City, Riyadh 12231, Saudi Arabia; qureshipmr@gmail.com (A.Z.Q.); drsamipmr@gmail.com (S.U.); nhalkeaid@gmail.com (N.H.A.); 3 Department of Physical Medicine and Rehabilitation, Combined Military Hospital Jhelum, Jhelum 49600, Pakistan; saeedbinayaz@gmail.com; 4 Department of Nursing, King Fahad Medical City, Riyadh 12231, Saudi Arabia; hmalotaibi@kfmc.med.sa

**Keywords:** spinal cord injuries, employment, rehabilitation, Saudi Arabia

## Abstract

Return to work is a challenging aspect of community integration for individuals with disabilities. The reintegration of individuals with spinal cord injury (SCI) is multifactorial; hence, regional challenges need to be investigated in the context of their clinical attributes and perceptions. A total of 121 male participants above 18 years of age with diagnosis of SCI and living at home were included in this cross-sectional survey. The study was conducted at a tertiary care rehabilitation facility in Saudi Arabia. The most common reported clinical barriers to employment were mobility, bladder incontinence, spasticity, musculoskeletal pain, and neuropathic pain. Bladder incontinence and musculoskeletal pain were the most common perceived clinical barriers for individuals with paraplegia and tetraplegia, respectively. A significant difference was observed for bowel incontinence as a reported barrier (*p* = 0.024) among adults less than thirty years of age in comparison with those older than thirty years. Spasticity as a barrier was reported more among patients who were older than thirty years (54.0%) compared to those younger than thirty years of age (37.9%) (*p* = 0.077). Twenty-two (23.7%) participants with paraplegia reported transfers as a perceived barrier to employment, which was significant (*p* = 0.014), and it was also reported as a significant barrier (*p* = 0.001) in individuals with tetraplegia (56%). This study shows that clinical conditions associated with SCI are considered potential barriers to employment by individuals with SCI. In terms of priority, the perceived barriers between individuals with tetraplegia and paraplegia were mostly different. This shows the need to consider relevant secondary health care conditions in goal setting while planning for employment in individuals with SCI.

## 1. Introduction

Patients with spinal cord injury (SCI) are at risk of developing a lifestyle with detrimental effects on physical fitness, social participation, and quality of life [1,2]. As a part of social empowerment, financial independence is one of the main considerations during the rehabilitation of persons with SCI, which is directly or indirectly linked with employment. Return to employment is positively associated with adjustment to disability, life satisfaction, and mental and physical health [3,4]. Despite this, the rate of employment after injury is reported to be 35–45% in the developed world, and it takes an average of 3.8 years to return to gainful employment [5,6,7,8,9,10,11]. It remains important to identify the barriers to employment in different health systems due to variations in lifestyle, socioeconomic factors, cultural attributes, and norms of the society.

In order to improve employment outcomes among individuals with SCI, a clear understanding of what factors influence employment outcomes is needed [9]. These factors vary and reflect the fact that employment outcomes are the result of a complex interaction between injury-related and contextual (personal/environmental) factors [5,8,9]. These factors can also be divided into modifiable and non-modifiable factors [9]. Generally, these barriers include level of education, type of employment, disability severity, age, time since injury, gender, marital status, social support, psychological problems, level of injury, severity of injury, vocational counseling, medical problems related to the SCI, employer role, environment, professional interests, motor FIM score and Barthel index [5,9,12,13]. One of the most frequently reported reasons for not working after SCI is the inability to fulfill the physical demands of the job (60%) [11,13]. SCI has a unique impact on bodily functions. Barriers to employment related to physical health in individuals with SCI include decreased mobility, spasticity, pain, pressure ulcers, incontinent bowel and bladder, workplace accessibility, and psychosocial issues [14]. It remains distinctively important to evaluate these clinical barriers in different populations to determine their social impact.

In Saudi Arabia, a disability survey in 2017 showed that the percentage of the Saudi population with disabilities accounted for 7.1% of the total Saudi population, with 3.7% males and 3.4% females [15]; however, there is no national registry for SCI in the country, and data regarding the percentage of individuals with SCI receiving rehabilitation services is lacking. Motor vehicle accidents are the most common cause of SCI in Saudi Arabia and account for 79–90% of all SCIs, predominantly involving males [16,17,18,19]. Though the female workforce is increasing in the country; it still remains low and males account for nearly 84% of the total labor workforce [20,21]. Another survey done on the same patient group showed that only a quarter of participants knew about vocational rehabilitation and nearly 96% of them reported not receiving any vocational rehabilitation services [22]. Though lack of awareness of vocational rehabilitation is one factor, barriers to employment in persons with SCI in the context of clinical or functional aspects are not reported in the country or Gulf region. The documentation of barriers to employment is important to empower the SCI population in the country for their vocational integration and devising relevant rehabilitation strategies. Though similar studies have been carried out in western countries and some low-middle income countries, the regional variations in the Gulf region and distinctiveness of social, cultural, and health system attributes render the need of exploring these aspects of SCI care locally. This study highlights the perceived clinical barriers to employment in males with SCI in Saudi Arabia, which are analyzed across age, marital status, and level of injury. The possible regional and institutional factors are further discussed in the relevant contexts.

## 2. Materials and Methods

A cross-sectional study commenced after approval from the ethics review committee. Adult males (>18 years of age) having SCI of any onset and living at home were included. Patients who had a concomitant brain injury or severe polytrauma and those having any other disabling mental or physical conditions (such as stroke, Parkinson’s disease, or Alzheimer’s disease) were excluded. The surveys were conducted using consecutive sampling. Out of 130 patients, 121 participants were included in the study who were following up in the SCI rehabilitation clinic of Rehabilitation Hospital at King Fahad Medical City Riyadh Saudi Arabia. Nine patients refused to participate. The facility is the largest ministry of health rehabilitation center offering inpatient and outpatient rehabilitation services across the country. Information was collected regarding age, marital status, level of injury (tetraplegia and paraplegia), and employment status. The age cut-off of 30 years was based on the labor market statistics, which reported that the highest number of Saudi male employees were in the age group between 30–34 years [20]. Demographic details, change in employment status related to injury, and awareness of vocational rehabilitation were reported for the same patient group in a previous report [22]. Patients were interviewed on perceived barriers to employment pertaining to physical health (spasticity, pressure ulcers, bowel incontinence, bladder incontinence, autonomic dysreflexia, neuropathic pain, musculoskeletal pain, transfers, mobility, accessibility, and psychological factors). The list of perceived barriers in the questionnaire was based on patient feedback, empirical evidence, and clinical categories used in the literature particular to SCI [5,6,13,14,23,24]. The questionnaire was finalized by an expert in the field of SCI. After pilot testing it on ten patients, it was given to participants with minor adjustments and was carried out by two physicians in the physical medicine and rehabilitation department who were fluent in Arabic and English. The survey is available in the Supplementary Materials.

### Statistical Analysis

Data were described as averages (mean ± SD) and percentages (frequency, %). Association of all the 11 perceived barriers was measured with Age, Marital status, and Level of injury by chi-square test. A *p*-value of <0.05 was considered significant. Statistical Package for Social Sciences (SPSS) v 21 (IBM SPSS Statistics, Armonk, NY, USA) and Microsoft Excel software 2016 (Microsoft Corporation, Redmond, WA, USA) were used for data analysis. The main outcome measure was to identify the health-related perceived barriers to employment in persons with SCI in Saudi Arabia.

## 3. Results

Demographics and clinical characteristics are shown in Table 1. The mean age of participants was 35.6 ± 14 years whereas the mean time since the onset of injury was 5.7 ± 3.85 years, with 44.6% of the patients having a period of 6 years and above since onset of injury.

Overall, the top five reported barriers to employment were mobility, bladder incontinence, spasticity, musculoskeletal pain and neuropathic pain (Table 2). Bladder incontinence and musculoskeletal pain were the most common perceived barriers for individuals with paraplegia and tetraplegia, respectively (Table 3).

A significant difference was observed for bowel incontinence as a reported barrier (*p* = 0.024) among adults less than thirty years of age in comparison with those older than thirty years. (Table 3). By contrast, spasticity as a barrier was reported more among patients who were older than thirty years (54.0%) as compared to those younger than thirty years of age (37.9%) (*p* = 0.077). Twenty-two (23.7%) participants with paraplegia reported transfers as a perceived barrier to employment, which was significant (*p* = 0.014), and it was also reported as a significant barrier (*p* = 0.001) in individuals with tetraplegia (56%). Similarly, 36 out of 53 participants who identified musculoskeletal pain as a barrier to employment had paraplegia, which was significant (*p* = 0.020) when compared with other barriers (Table 3). By and large, pressure ulcers and autonomic dysreflexia were the least common perceived clinical barriers to employment.

## 4. Discussion

Employment is one of the most important goals of individuals with SCI as it is associated with financial independence [1,2,3,4,5,6]. Neurological diseases manifest their functional impairments differently, offering unique challenges to vocational integration. SCI has specific factors related to physical health that can interfere with satisfactory vocational roles of individuals in the community; however, there are certain attributes that are not a direct outcome of SCI but are of particular relevance. One such non-modifiable factor is age. The mean age in our study population was 35.6 ± 13.9 years with nearly half (47.9%) less than 30 years of age. This is similar to previously published studies on SCI in Saudi Arabia [16,17,18,19,25,26]. In the general Saudi population, an overwhelming majority of the unemployed is between the age of 20 and 39, with the most unemployed youth between 20–24 years of age [27]. This poses an additional competitive challenge to individuals with SCI. Apart from age, male gender carries a particular significance in relation to SCI and employment in Saudi Arabia. First, males are predominantly involved in SCI in Saudi Arabia, as evidenced by the published literature [16,17,18,19,25,26]. Secondly, motor vehicle accidents remain the number one cause of SCI in the country, mostly involving males [16,17,18,19]. This is in relation to the fact that females were not allowed to drive until restrictions were practically lifted in 2018 [28], though females have been involved in traumatic SCIs, but not as drivers. Thirdly, the majority of the workforce in the country is male; with an unemployment rate of 5.6% and a labor force participation rate of 65.8% in 2020 [29]. Another contributor that makes employment crucial for males in Saudi Arabia is the marriage trends. The average age of first marriage in Saudi Arabia is 26.3 years for males and 21.8 years for females [30]. Most married males are between 35–39 years of age [30]. It is also not uncommon to have more than one wife. Considering that the level of unemployment in females is considerably higher compared to males [27], males are predominantly responsible for the financial income of their families. Considering these factors, SCI rehabilitation in Saudi Arabia is mainly faced with challenges pertaining mostly to males. This poses a collateral challenge for females with SCI in the community. The Human Resources Development Fund has taken initiatives to provide resources and facilitate individuals with SCI for employment and social empowerment for both males and females [31].

In previous studies, physical inability leading to difficult access to the workplace has been the most cited reason for failure to join any job. Krause et al. quoted a reason for not working as the inability to physically perform the same type of work after injury (60%) [32]. A systemic review inquired about barriers to employment and found that among the unemployed persons with SCI, 64% indicated mobility issues and lack of transportation to the workplace as being the main perceived barriers to employment [33]. Provision of reliable transportation was identified by many persons as an important predictor to return to work [33]. Other worth mentioning perceived barriers reported in the literature are psychosocial factors, pressure ulcers, pain, and lack of adequate education and assistive devices [33,34,35,36,37,38,39,40]. Some persons with SCI identified fear of biases held by the potential employers about their capabilities leading to limited employment prospects [34]. Others perceived apprehension of poor attitude of rehabilitation professionals and frequent hospitalizations, if they were injured during any work-related activity [33,35]. Few believed that exploring any job might deprive them of the monetary benefits they were receiving as disabled persons [36,37]. Inadequate education appropriate to the individual’s abilities, and lack of assistive devices, e.g., reachers, wheelchairs, and special keyboards, were stated as barriers in the studies by al Ghatit and Hanson, Arango-Lasprilla et al., Krause and Anson, Tsai et al., and Graham et al. [31,34,37,38,39]. Secondary health conditions, especially chronic pain and pressure ulcers, were reported as identified barriers by Tsai et al. and Matthew et al., respectively [39,40]. In our study, the majority of participants considered mobility issues (56.7%), incontinent bladder (55.4%), spasticity (46.3%) musculoskeletal pain (50.0%), and neuropathic pain (42.1%) as major hurdles to employment. Psychological factors, transfers, accessibility, and bowel incontinence were considered as barriers to employment by nearly one-third of the respondents. This demonstrates that, in addition to functionality, secondary health conditions are of considerable importance for individuals with SCI for employment.

Chronological age and associated factors have been found to be related to barriers to employment. In a study that examined the age cohorts of its sample, those in the older cohorts had a less optimistic view of returning to work, primarily because they were not physically capable of working compared to the younger cohort [40]. Older age of onset of SCI has also been found to be associated with additional barriers to employment, such as requiring additional physical support and decreased energy [41,42]. The need for more help can be attributed to several factors, including fatigue, muscle weakness, pain and stiffness, weight gain, and specific medical problems. The pain in the elderly population is generally musculoskeletal pain likely caused by wheelchair propulsion and transfers. In our study, age was significantly associated with two barriers to employment, namely spasticity and bowel incontinence. In the Arab world, the vast majority of the population is Muslim, and prayers in mosques or congregational prayers are a common practice, which makes personal hygiene a matter of considerable importance [43].

Marital status has been observed as a predictor of employment in some studies, [34,44,45,46] while in others it did not serve in a predictive manner for employment [47,48,49]. In many cultures, the spouse plays a considerable supportive role in situations that may arise due to health-related problems of family members. A young male, who otherwise may have the potential to be independent, may be confined to bed or home after an SCI. With no known prospects of holistic rehabilitation for such a patient, the wife or some other member of the family may take up the financial responsibilities. This may be due to lack of awareness, insufficient vocational resources and lack of opportunities for social empowerment for individuals with SCI. There can be an impact of social pressure and cultural obligations on partners or significant others. In our study, the majority of the patients were unmarried; however, similar to age, marital status had a significant association with bowel incontinence as a barrier for return to employment.

In this study, transfers and musculoskeletal pain were the barriers significantly associated with both levels of injury (paraplegia and tetraplegia). Comparing the percentages between paraplegia and tetraplegia, a greater percentage of patients with tetraplegia reported spasticity, neuropathic pain, bowel/bladder incontinence, transfers, psychological factors, mobility, and musculoskeletal pain as barriers to employment. (Table 3). Krause and Anson [31] reported that individuals with tetraplegia were more likely to complain about physical incapacity and lack of proper transportation as reasons behind unemployment, while individuals with paraplegia were more likely to indicate psychological problems as the contributing factor to unemployment. The differences in reporting of barriers depending upon the level of injury are not surprising. Due to increased motor deficits, individuals with tetraplegia have more problems with physical ability, mobility, and transportation. Similarly, individuals with tetraplegia may have more difficulty making emotional adaptations to their injuries. The published literature shows variable findings. The level of injury was not found to be related to employment by Krause [42] and Valtonen et al. [50] By contrast, Krause and Anson, [31] El Ghatit and Hanson, [34] Pflaum et al., [45] Castle, [51] and Wang et al. [52] reported that individuals with paraplegia had higher employment rates compared to individuals with tetraplegia.

Another finding to note in this study was the high percentage of retired people (31.4%) at the time of interview, despite the fact that the mean age in this study was 35.6 years. The apparent possibility could be the lack of vocational opportunities for individuals with disabilities and lack of awareness among patients and their families. The government of Saudi Arabia has realized this potentiality and has acceded to the United Nations Convention on the Rights of Persons with Disabilities with particular emphasis on article 27 [53]. The Saudi legislative measures emphasize that people with disabilities have the right to public employment. The Government of Saudi Arabia has also started different programs to support and empower the workforce of people with disabilities to work in the private sector, create a safe and supportive work environment for people with disabilities by adopting the best standards and practices in the field, and bridge the gap between business owners and job seekers [53].

### Study Limitations

This was a single-center cross-sectional study and lacks longitudinal follow-ups. A large, multicenter study is required to explore the employment-related challenges in individuals with SCI. In addition, this study was limited to males Since the local reporting on female SCI is rare, similar studies on females should be done, given the unique social-cultural aspects of female employment in Saudi Arabia. The questionnaire included limited barriers to employment; however, there are various other clinical and non-clinical factors that need to be explored in future studies to determine the magnitude of the problem and devise appropriate strategies. Similarly, perceived barriers based on type of injury (complete or incomplete SCI) were not recorded in our study.

## 5. Conclusions

Clinical conditions associated with SCI are considered potential barriers to employment by males with SCI in Saudi Arabia. The most common perceived barriers to employment among individuals with SCI were mobility, bladder incontinence, spasticity, musculoskeletal pain, and neuropathic pain. In terms of priority, the perceived barriers between individuals with tetraplegia and paraplegia were mostly different. This shows the need to consider relevant secondary health care conditions in goal setting while planning for employment of individuals with SCI.

## Figures and Tables

**Table 1:** Demographic and clinical characteristics of the participants (*n* = 121).

Variables	Mean ± SD (Min, Max)	Subgroups	*n* (%)
Age (years)	35.6 ± 13.9 (17, 87)		
		Age ≤ 30 years	58 (47.9)
		Age > 30 years	63 (52.1)
Time since Injury (years)	5.7 ± 3.85 (1, 21)		
		<2 years	15 (12.3)
		2–5 years	52 (42.9)
		6–10 years	41 (33.8)
		>10 years	13 (10.7)
Marital Status		Unmarried	75 (62.0)
		Married	46 (38.0)
Province of Residence		Northern	17 (14.1)
		Eastern	6 (5.0)
		Western	8 (6.7)
		Southern	30 (25.0)
		Central	60 (49.2)
Education Level		Illiterate or informal education	6 (5.0)
		Primary school	12 (9.9)
		Intermediate school	10 (8.3)
		Secondary school	56 (46.3)
		College or university degree	33 (27.3)
		Higher education	4 (3.3)
Level of Injury		Tetraplegia	27 (22.5)
		Paraplegia	94 (77.5)
Employment Status at the Time of Interview		Employed	20 (16.5)
		Unemployed	52 (43)
		Retired	38 (31.4)
		Student	11 (9.1)
Inpatient Rehabilitation Services		Received	105 (86.8)
		Not received	16 (13.2)
Vocational Rehabilitation Services		Received	5 (4.1)
		Not received	116 (95.9)

**Table 2:** Perceived clinical barriers to employment in a Saudi cohort with spinal cord injury.

Barrier		*n* (%)
Spasticity	No	65 (53.7)
	Yes	56 (46.3)
Neuropathic Pain	No	70 (57.9)
	Yes	51 (42.1)
Bowel Incontinence	No	81 (66.9)
	Yes	40 (33.1)
Bladder Incontinence	No	54 (44.6)
	Yes	67 (55.4)
Musculoskeletal Pain	No	68 (56.0)
	Yes	53 (50.0)
Pressure Ulcer	No	111 (93.3)
	Yes	8 (6.7)
Autonomic Dysreflexia	No	108 (90.0)
	Yes	12 (10.0)
Transfers	No	85 (70.8)
	Yes	35 (29.2)
Mobility	No	52 (43.3)
	Yes	68 (56.7)
Accessibility	No	79 (66.4)
	Yes	40 (33.6)
Psychological Factors	No	79 (65.3)
	Yes	42 (34.7)

**Table 3:** Perceived clinical barriers to employment after spinal cord injury in relation to age, marital status and level of injury.

Age (yr)					Marital Status				Level of Injury							
									Paraplegia				Tetraplegia			
Barrier		≤30	>30	*p* Value		Unmarried	Married	*p* Value		No	Yes	*p* Value		No	Yes	*p* Value
Spasticity	No	36 (62.1)	29 (46.0)	**0.077**	No	44 (58.7)	21 (45.7)	0.163	No	11 (40.7)	54 (57.4)	0.125	No	56 (57.1)	9 (39.1)	0.119
	Yes	22 (37.9)	34 (54.0)		Yes	31 (41.3)	25 (54.3)		Yes	16 (59.3)	40 (42.6)		Yes	42 (42.9)	14 (60.9)	
Neuropathic pain	No	34 (58.6)	36 (57.1)	0.869	No	48 (64.0)	22 (47.8)	0.080	No	12 (44.4)	58 (61.7)	0.109	No	59 (60.2)	11 (47.8)	0.279
	Yes	24 (41.4)	27 (42.9)		Yes	27 (36.0)	24 (52.2)		Yes	15 (55.6)	36 (38.3)		Yes	39 (39.8)	12 (52.2)	
Bowel incontinence	No	33 (56.9)	48 (76.2)	**0.024**	No	44 (58.7)	37 (80.4)	**0.013**	No	17 (63.0)	64 (68.1)	0.618	No	68 (69.4)	13 (56.5)	0.238
	Yes	25 (43.1)	15 (23.8)		Yes	31 (41.3)	9 (19.6)		Yes	10 (37.0)	30 (31.9)		Yes	30 (30.6)	10 (43.5)	
Bladder incontinence	No	21 (36.2)	33 (52.4)	0.074	No	32 (42.7)	22 (47.8)	0.579	No	13 (48.1)	41 (43.6)	0.676	No	43 (43.9)	11 (47.8)	0.732
	Yes	37 (63.8)	30 (47.6)		Yes	43 (57.3)	24 (52.2)		Yes	14 (51.9)	53 (56.4)		Yes	55 (56.1)	12 (52.2)	
Transfers	No	41 (71.9)	44 (69.8)	0.802	No	53 (71.6)	32 (69.6)	0.81	No	14 (51.9)	71 (76.3)	**0.014**	No	75 (77.3)	10 (43.5)	**0.001**
	Yes	16 (28.1)	19 (30.2)		Yes	21 (28.4)	14 (30.4)		Yes	13 (48.1)	22 (23.7)		Yes	22 (22.7)	13 (56.5)	
Accessibility	No	34 (59.6)	45 (72.6)	0.136	No	47 (63.5)	32 (71.1)	0.395	No	18 (69.2)	61 (65.6)	0.728	No	64 (66.0)	15 (68.2)	0.843
	Yes	23 (40.4)	17 (27.4)		Yes	27 (36.5)	13 (28.9)		Yes	8 (30.8)	32 (34.4)		Yes	33 (34.0)	7 (31.8)	
Pressure ulcer	No	53 (93.0)	58 (93.5)	0.902	No	69 (93.2)	42 (93.3)	0.985	No	25 (96.2)	86 (92.5)	0.508	No	90 (92.8)	21 (95.5)	0.651
	Yes	4 (7.0)	4 (6.5)		Yes	5 (6.8)	3 (6.7)		Yes	1 (3.8)	7 (7.5)		Yes	7 (7.2)	1 (4.5)	
Psychological factors	No	39 (67.2)	40 (63.5)	0.665	No	54 (72.0)	25 (54.3)	0.048	No	14 (51.9)	65 (69.1)	0.096	No	68 (69.4)	11 (47.8)	0.051
	Yes	19 (32.8)	23 (36.5)		Yes	21 (28.0)	21 (45.7)		Yes	13 (48.1)	29 (30.9)		Yes	30 (30.6)	12 (52.2)	
Mobility	No	23 (40.4)	29 (46.0)	0.531	No	29 (39.2)	23 (50.0)	0.245	No	9 (33.3)	43 (46.2)	0.234	No	45 (46.4)	7 (30.4)	0.165
	Yes	34 (59.6)	34 (54.0)		Yes	45 (60.8)	23 (50.0)		Yes	18 (66.7)	50 (53.8)		Yes	52 (53.6)	16 (69.6)	
Autonomic dysreflexia	No	50 (87.7)	58 (92.1)	0.428	No	68 (91.9)	40 (87.0)	0.381	No	22 (81.5)	86 (92.5)	0.094	No	90 (92.8)	18 (78.3)	0.037
	Yes	7 (12.3)	5 (7.9)		Yes	6 (8.1)	6 (13.0)		Yes	5 (18.5)	7 (7.5)		Yes	7 (7.2)	5 (21.7)	
Musculoskeletal pain	No	28 (56.0)	25 (44.6)	0.243	No	36 (54.5)	17 (42.5)	0.229	No	7 (29.2)	46 (56.1)	**0.020**	No	47 (54.7)	6 (30.0)	**0.047**
	Yes	22 (44.0)	31 (55.4)						Yes	17 (70.8)	36 (43.9)		Yes	39 (45.3)	14 (70.0)	

## Data Availability

The data that support the findings of this study are available from the corresponding author, [A.H.A.], upon reasonable request.

## Supplementary Materials

The following supporting information can be downloaded at: https://www.mdpi.com/article/10.3390/ijerph19084747/s1, File S1: Survey Questions.
