# Supplementary material for: Perceived Clinical Barriers to Employment for Males with Spinal Cord Injury in Saudi Arabia
Source: Int J Environ Res Public Health. 2022 Apr 14;19(8):4747. doi: 10.3390/ijerph19084747 (PMC9029978; doi:10.3390/ijerph19084747)
Supplement: Supplementary file 1 [file ijerph-19-04747-s001.zip › ijerph-1666212-supplementary.pdf]

Supplementary File S1

In your opinion what are the barriers of returning to work after sustaining spinal cord injury (Note: you can choose more than one answer)

- □ Spasticity
- □ Neuropathic pain e.g. numbness, burning, tingling, cutting sensation
- □ Incontinent bowel
- □ Incontinent bladder
- □ Transfers
- □ Accessibility to work place
- □ Pressure ulcer
- □ Psychological factor (depression, low mood, behavioral, emotional instability)
- □ Mobility
- □ Autonomic dysreflexia
- □ Musculoskeletal pain
